# Identification of MCM4 as a Prognostic Marker of Hepatocellular Carcinoma

**DOI:** 10.1155/2021/7479326

**Published:** 2021-12-18

**Authors:** Huandi Zhou, Le Jiang, Guohui Wang, Linlin Su, Liubing Hou, Xiaoying Xue

**Affiliations:** ^1^Department of Radiotherapy, Second Hospital of Hebei Medical University, Shijiazhuang, Hebei 050000, China; ^2^Department of Central Laboratory, Second Hospital of Hebei Medical University, Shijiazhuang, Hebei 050000, China; ^3^Center of Metabolic Diseases and Cancer Research (CMCR), Hebei Medical University, Shijiazhuang, Hebei 050000, China; ^4^Office of Academic Research, Second Hospital of Hebei Medical University, Shijiazhuang, Hebei 050000, China

## Abstract

**Methods:**

MCM4 expression difference in HCC were analyzed from TCGA and GEO data and verified by real-time PCR and western blot. ROC curve was used to analyze the diagnostic value of MCM4 and AFP. Additionally, the relationship between MCM4 and stage or nodal metastasis status or grade or age in TCGA cohort with HCC was observed from the UALCAN website. The univariate and multivariate Cox and functional analyses were done to explore the prognostic value of MCM4 in TCGA cohort.

**Results:**

It was found that MCM4 was significantly highly expressed in HCC tissues from TCGA, GEO, and experimental data. Furthermore, ROC curve analysis showed that MCM4 was superior to be a diagnostic biomarker than AFP from TCGA (AUC_MCM4_ = 0.9461, AUC_AFP_ = 0.7056) and GEO (GSE19665: AUC_MCM4_ = 0.8800, AUC_AFP_ = 0.5100; GSE64041 AUC_MCM4_ = 0.8038, AUC_AFP_ = 0.6304). AUC of MCM4 from real-time PCR result in 60 pairs of HCC and adjacent tissues was 0.7172, demonstrating the prediction value of MCM4. Besides, different expression tendencies of MCM4 among different stages or nodal metastasis status or grade or age were observed from the UALCAN website. In addition, multiROC analysis showed the advantage of MCM4 as a survival prediction at 1, 3, and 5 years with the higher AUC at 0.69 of 1 year, 0.65 of 3 years, and 0.61 of 5 years. It was shown that MCM4 was independently associated with OS in univariate and multivariate Cox analysis. And GSEA displayed that MCM4 was highly enriched in KEGG_CELL_CYCLE signaling pathway following higher correlation positively with CDC6, PLK1, CRC1, and BUB1B in HCC.

**Conclusion:**

MCM4 might be a potential biomarker in guiding the prognostic status of HCC patients.

## 1. Introduction

Hepatocellular carcinoma (HCC) is the sixth most common malignant tumor in the world, and its mortality ranks the third among all cancers. It is undoubtedly true that China is one of the high incidence areas of HCC in the world [1]. In China, there are as high as 54.7 people/100000 in the annual mortality of HCC patients, ranking the second [2]. HCC is characterized by short-onset cycle and rapid progress, which is not only difficult to cure but also easy to relapse. With the improvement of medical technology, the 5-year survival rate of HCC can reach 50%, but the high recurrence rate (>70%) seriously restricts the survival rate and prognosis of patients [3]. Therefore, looking for effective prognostic markers of HCC has become the key to early predict the risk of recurrence and metastasis of HCC and timely interventional treatment, so as to further improve the clinical therapeutic effect of hepatocellular carcinoma.

Minichromosomal maintenance 4 (MCM4) belongs to the family of MCM proteins (MCMs), a group of family proteins strongly linked with DNA replication and cell proliferation, participates in the regulation of DNA replication initiation, and can be used as an effective marker for tumor diagnosis [4]. In recent years, many studies have proved that MCMs have potential as prognostic markers in different tumors. It is closely related to the prognosis of prostate cancer, lung cancer, ovarian cancer, renal cancer, and bladder cancer with an increased expression of MCM2 [5]. It is the heralding of a poor prognosis of astrocytoma patients with high-level expression of MCM3 protein [6]. MCM5 and MCM6 have the potential as an independent prognostic marker of melanoma. Furthermore, MCM7 can also be used as a prognostic marker of colorectal cancer, lung cancer, and ovarian cancer [5]. In recent years, there is relatively lack of MCM4 research by comparison with a growing report about the researches of MCM2, MCM5, and MCM7 in systemic tumors. However, it has been found that MCM4 expression is related to other tumors. Kikuchi et al. [7] found that MCM4 may play a pivotal role in the proliferation of small cell lung cancer (SCLC) cells, which can be used as a therapeutic target for some patients with SCLC. Huang et al. [8] confirmed that the high expression of MCM4 in esophageal cancer was positively correlated with the pathological grade. A study [9] reported that some melanoma patients with the increased expression of MCM4 have a poor prognosis. The high expression level of MCM4 found by Kwok et al. [5] is related to the survival of patients with breast cancer, suggesting the potential of MCM4 as an independent prognostic marker for breast cancer patients. However, the role of MCM4 relevant to the prognosis of HCC has not yet been illustrated.

The Cancer Genome Atlas (TCGA) is a publicly funded project aimed at classifying and discovering major genomic changes leading to cancer, so as to create a Comprehensive Cancer Genome Atlas. So far, large samples of more than 30 human tumors have been analyzed by TCGA researchers through large-scale genome sequencing and comprehensive multidimensional analysis. More and more key cancer factors were found by bioinformatics analysis based on TCGA database, which provides ideas and targets for improving cancer diagnosis and treatment standards [10].

In this research, based on the bioinformatics analysis from TCGA and GEO database and experiment verification, we implored the relationship between MCM4 and the HCC diagnosis and prognosis, which indicated MCM4 might be helpful in predicting HCC patients' prognosis status.

## 2. Methods and Materials

### 2.1. Data Source

**Figure 1:**
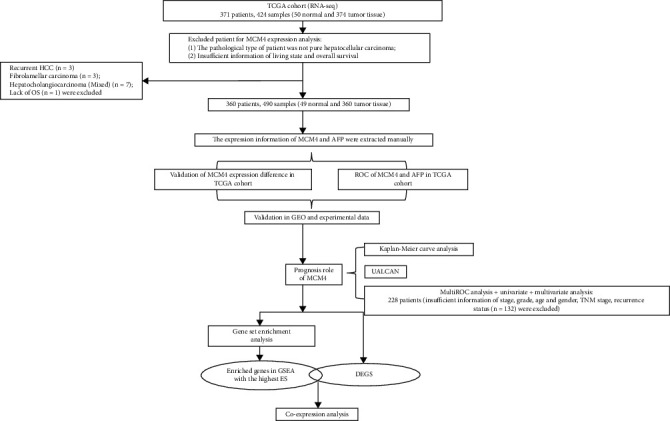
Flowchart of the whole study. Abbreviations: HCC: hepatocellular carcinoma; TCGA: The Cancer Genome Atlas; GEO: Gene Expression Omnibus; ROC: receiver operating characteristic; DEGs: differentially expressed genes; GSEA: Gene Set Enrichment Analysis.

The mRNA expression profiles and the corresponding clinical information from HCC patients were downloaded from TCGA dataset (https://portal.gdc.cancer.gov/) in December 8, 2020, which was calculated on an Illumina HiSeq RNA-sequencing platform and publicly available and open access, containing 360 primary hepatocellular carcinoma HCC tissues and 49 adjacent nontumorous liver tissues. Due to the requirement to the data integrality, patients who met the following criteria were excluded from subsequent analysis: (1) the pathological type of patient was not primary hepatocellular carcinoma (*n* = 3); (2) the pathological type of patient was not pure hepatocellular carcinoma, such as fibrolamellar carcinoma (*n* = 3) and hepatocholangiocarcinoma (mixed) (*n* = 7); (3) insufficient information of living state and overall survival (*n* = 1) for survival analysis; and (4) insufficient information of age, gender, grade, stage, TNM stage, and recurrence status (*n* = 132) for univariate analysis, multivariate analysis, and multiROC analysis. Finally, 360 tumor samples from different individuals and 49 adjacent nontumorous samples were selected in this study. Consequently, the patients (*n* = 360) were further subjected to survival and ROC analysis, and the patients (*n* = 228) were further subjected to univariate/multivariate analysis and multiROC analysis. The detailed process is shown in Figure 1. Besides, the two datasets GSE64041, containing 60 tumor tissue expression information and 65 nontumor liver tissue expression information, and GSE19665 including 10 pairs of tumor and adjacent tissue expression information were obtained from Gene Expression Omnibus (GEO) (website: http://www.ncbi.nlm.nih.gov/geo/) for expression and ROC analysis.

### 2.2. Protein Expression by Western Blot

As previously described [11], cells were washed with ice-cold PBS twice for 5 min/time and lysed with RIPA (P1300B, Beyotime, Shanghai, China) and protein phosphatase inhibitors (Solarbio). Then, the samples were equilibrated and denatured with 5× loading buffer for 100°C for 10 min. Protein concentrations were measured using BCA kit (PC0020, Solarbio, Shanghai, China). 20 *μ*g of protein was separated on 10% SDS-PAGE, transferred onto a PVDF membrane (Millipore), blocked with 3% nonfat milk in TBST (Tris base Tris-buffered saline and 0.1% Triton-100, pH 7.4). Membranes were incubated with primary antibodies for MCM4 (1 : 500, sc-28317, Santa Cruz, USA) and *β*-actin (1 : 2000, TA-09, ZSGB-BIO, Beijing, China) overnight at 4°C. After washing three times with 1× TBST, they were incubated with secondary antibodies for an hour at room temperature. Again, they were washed three times with 1× TBST. Proteins were visualized by enhanced chemiluminescence (BL520A, Biosharp, Beijing, China) and autoradiography. ImageJ was used to analyze the density of the bands.

### 2.3. RNA Isolation and Real-Time PCR

60 pairs of HCC patients and paracancerous tissues were collected. For the study, the patients' informed consent (verbal) was obtained, and the subject and the study were approved by the Ethics Committee of Second Hospital of Hebei Medical University (2021-R044). These tissues were fully lysed in TRIzol reagent (Invitrogen) by a homogenizer, and total RNA was extracted using TRIzol according to the manufacturer's protocol. After concentration measurement by NanoDrop 2000, 1 *μ*g total RNA was used for reverse transcription in a final volume of 20 *μ*l with reverse transcriptase (Thermo Fisher) according to the manufacturer's instructions. cDNA was used for the quantification of mRNA by real-time PCR using the All-in-One™ qPCR (QP003, FulenGen, Guangzhou, China). The reaction system (20 *μ*l) contained the corresponding cDNA, forward and reverse primers, and SYBR-Green PCR Master Mix. The design formula of fold changes was the 2^−[(Ct of target gene) − (Ct of *β*-actin)]^ method. The specific primers of MCM4 and *β*-actin are as follows: MCM4 forward: CAGCAGCAAATCCCATTGAGT; MCM4 reverse: TGTCATAG GCTTCGTCCTGAG; *β*-actin forward: CATGTACGTTGCTATCCAGGC; *β*-actin reverse: CTCCTTAATGTCACGCACGAT.

### 2.4. MCM4 Expression Analysis of HCC

Gene raw expression data from TCGA were normalized, and Entrez IDs from both of TCGA and GEO were converted to gene IDs by using SangerBox (http://sangerbox.com/). The expression of MCM4 was manually extracted from TCGA and GEO dataset and compared in tumor and nontumor groups.

### 2.5. Validation of MCM4 Prognostic Signature

To validate the pivotal role of MCM4 on HCC prognosis, the patients were stratified into a low-expression group and high-expression group bounded by a lower quartile of MCM4. The Kaplan-Meier (KM) survival analysis with log-rank test and receiver operating characteristic (ROC) curve and multiROC analysis were used to validate the MCM4 prognostic signature.

### 2.6. Clinical Correlation Analysis Using the UALCAN Database

UALCAN (http://ualcan.path.uab.edu/index.html) is an interactive web-based tool to perform analyses of gene expression data from TCGA [12]. The UALCAN database was used to analyze the expression levels of MCM4 in liver hepatocellular carcinoma (LIHC) based on individual cancer stages, nodal metastasis status, tumor grade, patient's age, and patient's gender.

### 2.7. Gene Set Enrichment Analysis

Gene Set Enrichment Analysis (GSEA) was performed to explore the potential biological pathways. Based on the lower quartile expression of MCM4, the whole set of 360 HCC samples was divided into two groups. Then, GSEA software (GSEA_4.1.0, http://software.broadhttp://institute.org/gsea/) was conducted on JAVA 8.0 platform. The annotated gene sets h.all.v7.2.symbols.gmt and c2.cp.kegg.v7.2.symbols.gmt obtained from the GSEA official website (http://www.gsea-msigdb.org/gsea/index.jsp) were chosen as the reference set to calculate enrichment score (ES) which estimated whether genes from prior defined gene set are enriched in the high-/low-expression group of MCM4 or not. The number of permutations was set to 1000. Gene size smaller than 15 or larger than 500 was excluded. A gene set was considered an enriched group when the normalized *p* value < 0.05 and FDR score < 0.05.

### 2.8. Coexpression Analysis

The patients were sorted into the low-expression group and high-expression group bounded by a lower quartile of MCM4. The “limma” package of R software was used to screen differentially expressed genes (DEGs) that were coexpressed with MCM4. DEGs including significantly upregulated and downregulated genes were screened to subsequent analysis with an adjusted *p* value < 0.05 and absolute log2 fold change (FC) > 1. In addition, the pheatmap package was used to plot the first 20 upregulated genes and the first 20 downregulated genes associated with MCM4. And the intersection genes between genes in KEGG_CELL_CYCLE biological pathway enriched by GSEA and the DEGs with log2FC > 1 were analyzed further for coexpression with MCM4 with a correlation coefficient > 0.7 and *p* < 0.001. The Corrplot and Circlize packages were used to generate a circular plot of the top genes associated with MCM4.

### 2.9. Statistical Analysis

Statistical analyses were conducted with the GraphPad Prism 8.0 software (GraphPad, Inc., La Jolla, CA, USA). Cox proportional hazard regression model was used for univariate or multivariate analysis to explore MCM4 independent prognostic role. The overall survival was analyzed with the Kaplan-Meier method, using the log-rank test to determine the difference. *p* values for each analysis are marked on figures, and the level of statistical significance was defined as *p* < 0.05 (^∗^*p* < 0.05; ^∗∗^*p* < 0.01; ^∗∗∗^*p* < 0.001).

## 3. Results

### 3.1. MCM4 Is Highly Expressed in Hepatocellular Carcinoma

**Figure 2:**
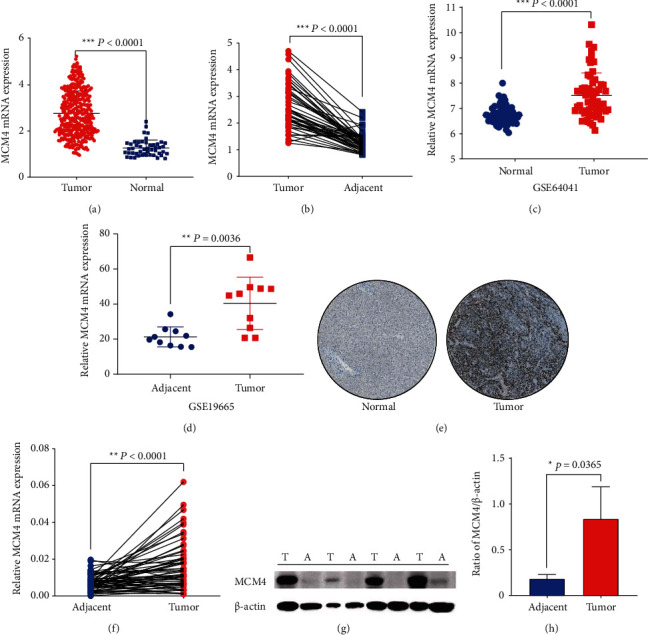
MCM4 is high expression in hepatocellular carcinoma. (a–d) MCM4 was significantly upregulated in HCC from 360 HCC tissues and 49 normal tissue expression profile in TCGA (A), 49 pairs of HCC tissues and adjacent tissue expression profile in TCGA (b), GSE64041 data (c), and GSE 19665 data (d); (e) protein levels of MCM4 in normal tissue (left) and tumor tissue (right) by immunohistochemistry based on the Human Protein Atlas (liver normal tissue, patient ID 1846: female; 32 years old, hepatocyte staining intensity: weak; quantity: <25%; location: nuclear; liver cancer tissue, patient ID 983: female, 53 year, intensity: strong; quantity: >75%; location: nuclear); (f) the expression of MCM4 in 60 pairs of HCC and adjacent tissues from frozen specimens tested by real-time PCR; (g, h) the expression of MCM4 in 4 pairs of HCC and adjacent tissues from frozen specimens tested by western blot (g) and the statistical result of western blot (h). T: tumor; A: adjacent.

To explore MCM4 expression level in hepatocellular carcinoma, the MCM4 expression information from TCGA data was extracted. After screening based on the exclusion criteria (Figure 1), the nontumor samples or tumor samples with data deficiency were excluded, MCM4 expression information from 360 HCC tissues and 49 normal tissues was retrieved from TCGA database. Finally, 228 HCC patients and their clinical information were downloaded for prognosis analysis in this study. Compared with normal adjacent tissues, MCM4 was positively upregulated in HCC tissues (Figure 2(a), *p* < 0.0001). Furthermore, comparing MCM4 expression between 49 pairs of HCC tissues and adjacent tissues, a significant upregulation of MCM4 was detected in tumor tissues (Figure 2(b), *p* < 0.0001). Additionally, a total of 60 HCC tumor tissue and 65 non-tumor liver tissue data in the GSE64041 dataset (*p* < 0.0001) and 10 pairs of tumor and adjacent tissue data in GSE19665 (*p* = 0.0036) were downloaded. Consistent with the result in TCGA, there was evident high expression of MCM4 in HCC tissues (Figures 2(c) and 2(d)). At the same time, the experiment data from 60 pairs of HCC patients and paracancerous tissues was validated again for the abnormal expression phenomenon through real-time PCR (*p* < 0.0001, Figure 2(f)). In addition, the protein level of MCM4 was significantly higher in HCC tissues (high, >75%, location: nuclear, patient ID 983) compared with normal tissues (weak, <25%, location: nuclear, patient ID 1846) based on Human Protein Atlas (HPA) [13] (Figure 2(e)) and western blot validation from 4 pairs of tissues of HCC patients (Figures 2(g) and 2(h), *p* = 0.0365).

### 3.2. The ROC Curve Indicates a Good Performance of MCM4 for the Diagnosis of HCC Patients

**Figure 3:**
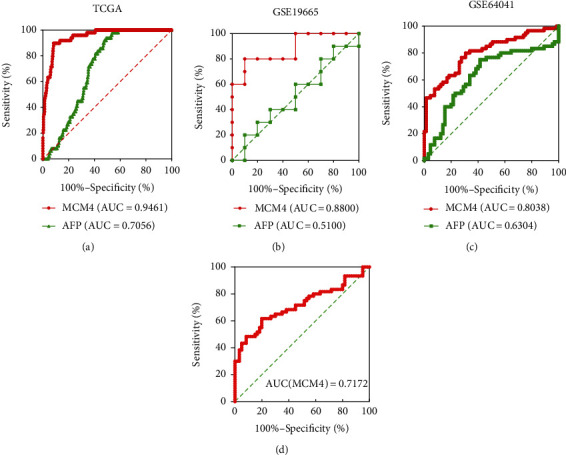
ROC curve analysis of MCM4 for the diagnosis of HCC patient: (a) ROC curve analysis of MCM4 and AFP based on TCGA HCC data; (b) ROC curve analysis MCM4 and AFP based on GSE19665 data; (c) ROC curve analysis MCM4 and AFP based on GSE64041 data; (d) ROC curve analysis MCM4 based on real-time PCR experimental data in 60 pairs of HCC and adjacent tissues.

As everyone knew, serum alpha fetoprotein (AFP), as a carcinoembryonic glycoprotein, was the first to be studied as a biomarker of hepatocellular carcinoma by Abelev in 1968 [14]. AFP is the most commonly used detection index in the clinical diagnosis of early hepatocellular carcinoma. However, it is not satisfactory that AFP is a biomarker in the early diagnosis of HCC, because of the limited sensitivity and specificity of AFP in the diagnosis of hepatocellular carcinoma with some false negative and false positive, which greatly limits the clinical application prospect of AFP in monitoring HCC. According to the MCM4 and AFP expression levels from 360 HCC tumor tissues and 49 adjacent tissue data in TCGA, as an HCC diagnosis biomarker, MCM4 got a higher AUC of ROC curve than AFP, which showed better sensitivity and specificity (AUC(MCM4) = 0.9461, AUC(AFP) = 0.7056, Figure 3(a)). To evaluate the predictive value of MCM4 in the diagnosis of HCC patients in other datasets, the ROC curve analysis was assessed in the GEO microarray data (GSE19665 and GSE64041). It showed the same advantage on the diagnosis of HCC than AFP (GSE19665: AUC(MCM4) = 0.8800, AUC(AFP) = 0.5100; GSE64041: AUC(MCM4) = 0.8038, AUC(AFP) = 0.6304, Figures 3(b) and 3(c)). Besides, it was indicated that MCM4 was a potential HCC diagnosis biomarker verified by ROC curve analysis based on real-time PCR experiment data from the 60 pairs of HCC tissues and adjacent tissues (AUC(MCM4) = 0.7172, Figure 3(d)).

### 3.3. Overexpression of MCM4 Correlated with Poor Prognosis of HCC and Clinical Parameters in HCC Patients

**Figure 4:**
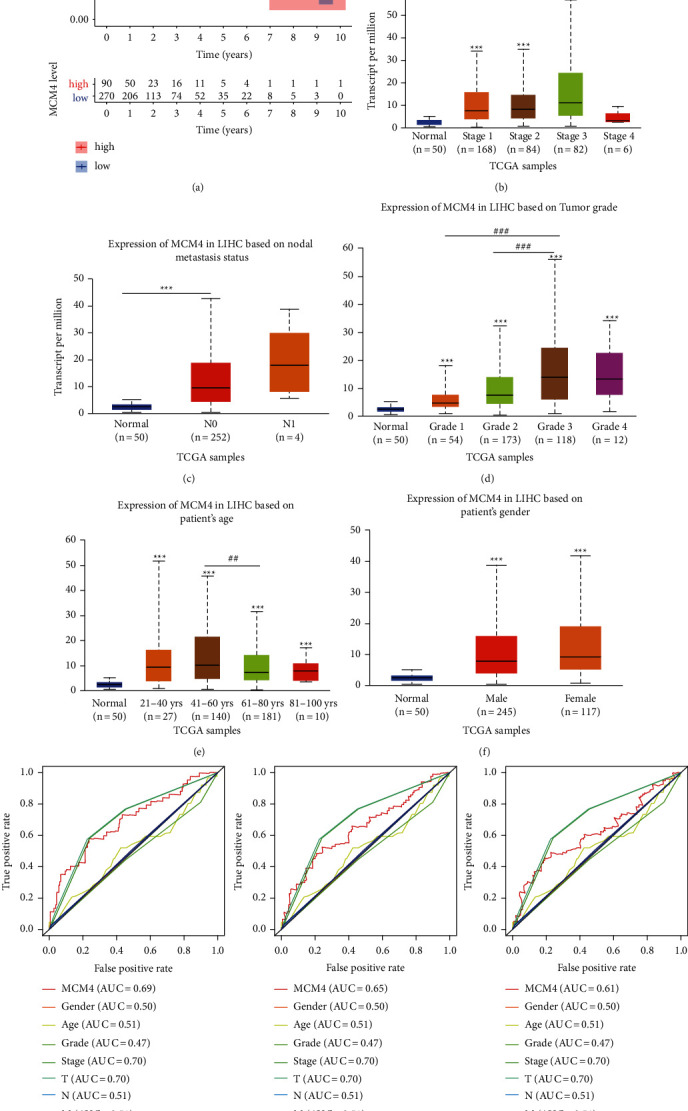
Overexpression of MCM4 correlated with poor prognosis of HCC and clinical parameters in HCC patients. (a) The K-M survival curves show the OS based on the relatively high- and low-expression patients divided by a lower quartile of MCM4. (b–f) Expression of MCM4 in different stages (b), nodal metastasis status (c), grade (d), age (e), and gender (f) of HCC; (g) multiROC curve analyzes the survival prediction for MCM4, AFP, gender, age, stage, TNM stage, grade, and recurrence status at 1-, 3-, and 5-year OS.

To clarify the prognostic role of MCM4, we first analyzed the correlation between the expression of MCM4 and the overall survival (OS) in human HCC samples from TCGA data. After screening, 228 HCC patients with sufficient clinical information of age, gender, stage, TNM, grade, vital status, and recurrence status were selected for prognosis analysis in this study. The detailed clinical information classification and percentages are shown in Table 1. According to the expression levels of MCM4, the patients were classified into the low-expression group and high-expression group. KM survival analysis with log-rank test showed that HCC patients who had a higher expression of MCM4 signified a shorter OS of the patients in comparison with the low-expression group (*p* < 0.001, Figure 4(a)). Besides, we evaluated its expression in HCC patients included in TCGA database using the UALCAN portal. There were different expression tendencies of MCM4 among different stages or nodal metastasis status or grade or age, although some difference has no significance. And no difference of MCM4 expression on gender was found (Figures 4(b)–4(f)). In addition, multiROC analysis showed the survival prediction for MCM4, AFP, gender, age, stage, TNM stage, grade, and recurrence status at 1, 3, and 5 years, which could be a further proof for a better prognosis biomarker of MCM4 in HCC with high AUC at 0.69 of 1 year, 0.65 of 3 years, and 0.61 of 5 years (Figure 4(g)).

### 3.4. MCM4 Is an Independent Risk Clinical Factor for HCC

**Figure 5:**
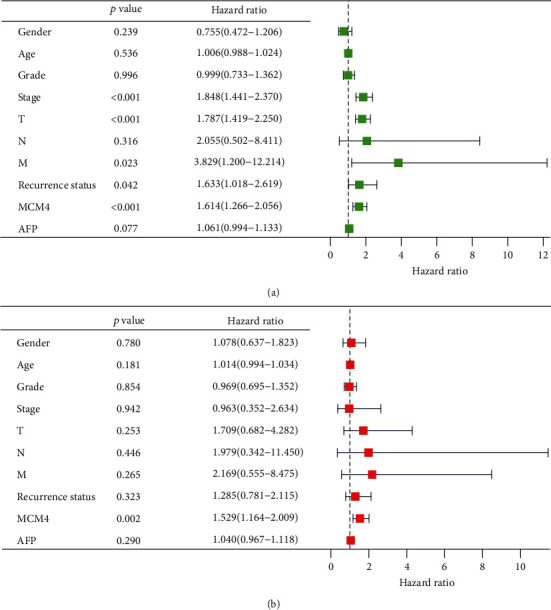
Univariate and multivariate Cox analysis of the correlation MCM4 with OS in patients with HCC. (a) Univariate analysis analyzes the association between MCM4 and OS. (b) Multivariate Cox analysis analyzes the association between MCM4 and OS.

The univariate and multivariate Cox proportional hazards regression methods were executed to evaluate the independent predictive value of MCM4 and clinical parameters, such as gender, age, stage, grade, and AFP, in 334 HCC patients with sufficient clinical information from TCGA data. Univariate Cox analysis showed that MCM4 (HR = 1.614; 95%CI = 1.266-2.056; *p* < 0.001), stage (HR = 1.848; 95%CI = 1.441-2.370; *p* < 0.001), T stage (HR = 1.787; 95%CI = 1.419-2.250; *p* < 0.001), and recurrence status (HR = 1.633; 95%CI = 1.018-2.619; *p* = 0.042) were high-risk factors, while gender, age, grade, N stage, M stage, and AFP did not correlate with OS (Figure 5(a)). Multivariate Cox analysis showed that only MCM4 (HR = 1.529; 95%CI = 1.164-2.009; *p* = 0.002) was independently associated with OS, which suggested that MCM4 could be an independent prognostic indicator for HCC. (Figure 5(b)).

### 3.5. Gene Set Enrichment Analysis of MCM4 and Coexpression Analysis of MCM4

**Figure 6:**
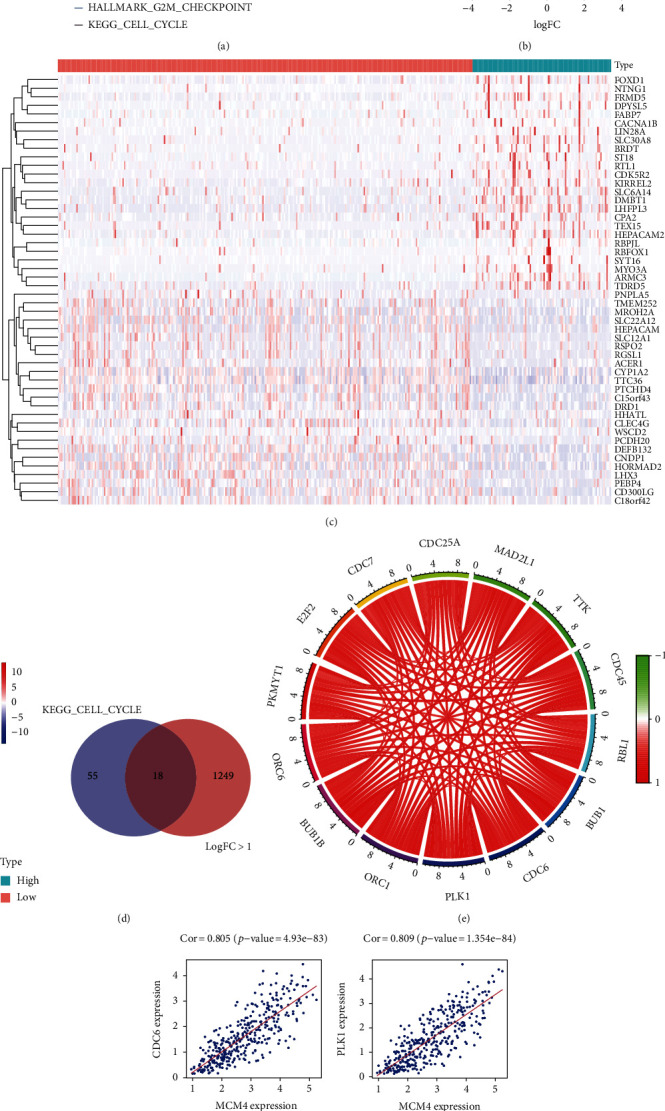
Gene Set Enrichment Analysis and coexpression analysis of MCM4 in TCGA HCC dataset. (a) GSEA of MCM4 using TCGA database; (b) volcano plot shows DEGs between the low-expression group and high-expression group (∣logFC | >1 and adjusted *p* value < 0.05); (c) heatmap of the top 20 genes positively and the top 20 genes negatively associated with MCM4; (d) Venn diagram between genes enriched in KEGG_CELL_CYCLE signaling pathway and DEGs (∣logFC | >1 and adjusted *p* value < 0.05); (e) circular plot of the top 14 genes related to the MCM4 gene; (f) scatter plot of the top four genes related to the MCM4 gene.

To identify the hallmark and KEGG signaling pathways in HCC between low- and high-MCM4-expression groups, GSEA was employed based on TCGA data. It showed significant differences (*p* value < 0.05 and FDR score < 0.05) in enrichment using an annotated gene set (h.all. v7.2. symbols and c2.cp.kegg. v7.2. symbols). As shown in Figure 6(a) HALLMARK_DNA_REPAIR, HALLMARK_E2F_TARGETS, HALLMARK_G2M_CHECKPOINT, and KEGG_CELL_ CYCLE were enriched in the MCM4 high-expression phenotype. In addition, to explore the DEGs that were coexpressed with MCM4, the “limma” package of R software was used to analyze the DEGs in two groups divided by a lower quartile of MCM4. DEGs including significantly upregulated and downregulated genes were screened to subsequent analysis with an adjusted *p* value < 0.05 and absolute log2 fold change (FC) > 1 (Figure 6(b)). 1267 DEGs were found between the high-expression group and low-expression group, and a heatmap of the first 20 upregulated genes and the first 20 downregulated is shown in Figure 6(c). Based on a normalized enrichment score (NES) of GSEA, KEGG_CELL_CYCLE was selected as the most significantly enriched hallmark and KEGG signaling pathway. The intersection genes (18 genes) between genes enriched in KEGG_CELL_CYCLE (73 genes) and DEGs (1267 genes) were selected to analyze the correlation with MCM4. It was shown that MCM4 was remarkably positively associated with ORC6, PKMYT1, E2F2, CDC7, CDC25A, MAD2L1, TTK, CDC45, RBL1, BUB1, CDC6, PLK1, CRC1, and BUB1B with a higher correlation coefficient than 0.7, which was exhibited in a circular plot (Table 2, Figure 6(e)). Additionally, the top four genes with a higher correlation coefficient than 0.7 is shown in the scatter plot (Figure 6(f)).

## 4. Discussion

Hepatocellular carcinoma, one of the most frequently diagnosed malignancies, is associated with a significant mortality rate [15]. Due to the high incidence of metastasis and recurrence, patients with HCC, especially at advanced stages, usually had a poor prognosis [16]. Although there is rapid development of medical technology in recent years, such as interventional therapy, curative resection, or liver transplantation to targeted therapy or immunotherapy, the outcomes of HCC are still undesirable [3]. Therefore, there is an urgent need to explore and identify new molecular markers that could predict HCC patient prognosis more accurately.

As everyone knew, AFP, as a carcinoembryonic glycoprotein, was first to be studied as a biomarker of hepatocellular carcinoma by Abelev in 1968 [14]. So far, AFP is the best indicator for the early diagnosis of HCC. Although AFP has important value for postoperative monitoring, its specificity needs to be improved. Under different critical values, the sensitivity of AFP detection is 40%~65%, and the specificity is 76%~96%, with some false negative and false positive, which greatly limits the clinical application prospect of AFP monitoring hepatocellular carcinoma [17].

MCM4 acts as the component of the minichromosome maintenance family (MCM) which contains six highly related MCM genes (MCM2-7) and participates in DNA replication initiation and elongation in eukaryotic cells [18]. Numbers of researches have reported that MCM genes played essential roles in various tumors [19, 20]. Han et al.'s team noticed a significant role for MCM4 overexpression in human laryngeal squamous cell carcinoma (LSCC) tissues and found that MCM4 overexpression is a potential prognostic marker for LSCC [21]. Long et al. identified 14 core genes (PKMYT1, TTK, CHEK1, CDC20, PTTG1, MCM2, CDC25C, MCM4, CCNB1, CDC45, MAD2L1, CCNB2, BUB1, and CCNA2) that are important for lung adenocarcinoma (LUAD) by bioinformatics analysis and may be potential therapeutic targets [22]. Byun et al. reported that ohmyungsamycin A (Compound 1) upregulated the expression of the CDK inhibitor p27 but downregulated the expression of Skp2 and MCM4, then induced G0/G1 cell cycle in human colorectal cancer cells [23]. But, comprehensive analyses of the diagnostic values, especially for prognostic values of MCM4 genes all alone in HCC, remain to be elucidated.

In this article, MCM4, a member of a family of proteins closely related to DNA replication and cell proliferation, was selected as a potential biomarker of HCC prognosis. First of all, sequential data extract was performed from TCGA and GEO database, which resulted in verification of the key gene MCM4 (Figures 1(a)–1(d)). Then, the experiment data from real-time PCR in 60 pairs of HCC tissues and adjacent tissues further demonstrated the remarkable high expression status of MCM4 in the tumor group (Figure 1(f)). More than that, the results from HPA IHC data and western blot detection of 4 pairs of HCC patients' tissues identified MCM4 protein high expression level in the liver cancer group (Figures 1(e), 1(g), and 1(h)).

In recent years, more and more biomarkers of HCC were found based on bioinformation [24, 25]. But there were few researches compared with the classic diagnosis or prognosis biomarker, AFP. Yang et al. [25] reported that the SFN and SPP1 function as oncogenes in HCC, which correlates with tumor grade and poor survival in HCC based on bioinformation. Zhang et al. [24] found the prognostic value of NuRD complex expression in HCC using the RNA-seq data obtained from TCGA project. Both of them did not compare the efficiency as biomarker of HCC. In this article, it is worth noting that we compared the diagnostic value of MCM4 and AFP though ROC analysis based on TCGA data and two GEO datasets (GSE19665 and GSE64041), which exhibited the superiority of MCM4 as a diagnostic biomarker in comparison with AFP (Figure 3). The AUC of MCM4 from TCGA data was 0.9461, while the AUC of AFP was 0.7056. In the GEO database, AUC(MCM4) and AUC(AFP) from GSE19665 were 0.8800 and 0.5100, respectively. AUC (MCM4) and AUC(AFP) from GSE64041 were 0.8308 and 0.6304, respectively. As for prognostic biomarker, it was as well as showing that MCM4 was in an advantageous position than AFP. MultiROC analysis on MCM4, AFP, gender, age, stage, TNM, grade, and recurrence status at survival prediction of 1 year, 3 years, and 5 years showed that stage and T stage were with the highest AUC, 0.70 at 1, 3, and 5 years, and MCM4 displayed the second highest AUC, 0.69 at 1 year, 0.65 at 3 years, and 0.61 at 5 years (Figure 4(g)). Moreover, univariate and multivariate Cox analysis from 228 HCC patients in TCGA data proved that MCM4 was an independent hazardous factor for HCC patients' survival (univariate Cox: HR = 1.614, 95%CI = 1.266-2.056, *p* < 0.001; multivariate Cox: HR = 1.529, 95%CI = 1.164-2.009, *p* = 0.002). However, there was lack of statistical significance that AFP is a prediction OS biomarker of HCC (univariate Cox: HR = 1.061, 95%CI = 0.994-1.133, *p* = 0.077; multivariate Cox: HR = 1.040, 95%CI = 0.967-1.060, *p* = 1.118).

Additionally, the results of GSEA speculated that the high levels of MCM4 might be involved in HCC progression by regulating HALLMARK_DNA_REPAIR, HALLMARK_E2F_TARGETS, HALLMARK_G2M_ CHECKPOINT, and KEGG_CELL_CYCLE. Finally, coexpression analysis demonstrated that MCM4 was remarkably associated with CDC6, PLK1, CRC1, and BUB1B in KEGG_CELL_CYCLE signaling pathway, which suggests that MCM4 might affect HCC prognosis by regulating HCC cell cycle. There were few studies focused on the association between MCM4 and HCC, which suggests that this study may provide an idea for the development of HCC treatment strategies in the future. However, it lacks a large number of samples for clinical verification. The mechanism of its expression regulation still needs more exploration to identify.

## 5. Conclusion

In conclusion, this study investigated the relationship between MCM4 and hepatocellular carcinoma prognosis. All results above proved that high expression of MCM4 was correlated with worse prognosis, and MCM4 was an independent high-risk prognostic indicator for patients with HCC.

## Figures and Tables

**Table 1:** The clinical characteristics of patients with HCC in TCGA.

Characteristics	*n*	%
Adjacent noncancerous tissue	49	
Hepatocellular carcinoma tissue	360	
Age (years)		
≤60	173	48.06
>60	187	51.94
Gender		
Male	243	67.50
Female	117	32.50
Stage		
I	167	46.39
II	81	22.50
III	84	23.33
IV	4	1.11
NA	24	6.67
Tumor size		
T1–T2	265	73.61
T3-T4	92	25.56
NA	3	0.83
Tumor node		
N0	245	68.06
N1	3	0.83
NA	112	31.11
Tumor metastasis		
M0	259	71.94
M1	4	1.11
NA	97	26.94
Grade		
G1	53	14.72
G2	171	47.50
G3	120	33.33
G4	11	3.06
NA	5	1.39
Vital status		
Live	231	64.17
Dead	129	35.83
Recurrence status		
Yes	168	46.67
No	184	51.11
New primary tumor	8	2.22

**Table 2:** The correlation analysis between MCM4 and DEGs.

Gene	Cor	*p*
ORC6	0.706	1.11753*E*-55
PKMYT1	0.722	3.7411*E*-59
E2F2	0.748	1.06032*E*-65
CDC7	0.754	2.04734*E*-67
CDC25A	0.755	1.62422*E*-67
MAD2L1	0.766	1.30159*E*-70
TTK	0.767	6.60321*E*-71
CDC45	0.781	3.30173*E*-75
RBL1	0.787	3.63849*E*-77
BUB1	0.794	2.1286*E*-79
CDC6	0.805	4.92994*E*-83
PLK1	0.809	1.35369*E*-84
ORC1	0.809	1.82908*E*-84
BUB1B	0.813	2.77714*E*-86

Abbreviations: Cor: the correlation coefficient of Pearson analysis; DEGs: differentially expressed genes; ORC6: origin recognition complex subunit 6; PKMYT1: protein kinase, membrane associated tyrosine/threonine 1; E2F2: E2F transcription factor 2; CDC7: cell division cycle 7; CDC25A: cell division cycle 25A; MAD2L1: mitotic arrest deficient 2 like 1; TTK: TTK protein kinase; CDC45: cell division cycle 45; RBL1: RB transcriptional corepressor like 1; BUB1: BUB1 mitotic checkpoint serine/threonine kinase; CDC6: cell division cycle 6; PLK1: polo like kinase 1; ORC1: origin recognition complex subunit 1; BUB1B: BUB1 mitotic checkpoint serine/threonine kinase B.

## Data Availability

The datasets generated and/or analyzed during the current study are presented in the main file. Additional data are available from the corresponding author on reasonable request.
